# Pre-clinical toxicity assessment of *Artemisia absinthium* extract-loaded polymeric nanoparticles associated with their oral administration

**DOI:** 10.3389/fphar.2023.1196842

**Published:** 2023-07-10

**Authors:** Sana Kauser, Mohd Mughees, Sanskriti Swami, Saima Wajid

**Affiliations:** Department of Biotechnology, School of Chemical and Life Sciences, Jamia Hamdard, New Delhi, India

**Keywords:** *Artemisia absinthium*, N-isopropyl acrylamide, N-vinyl pyrrolidone, acrylic acid, polymeric nanoparticles, LD50, acute oral toxicity, OECD guidelines 423

## Abstract

**Background:** This study was designed to quantify the composition of the ethanolic extract of *Artemisia absinthium* through gas chromatography–mass spectrometry analysis and ensure *in vivo* safety of *A. absinthium* extract-loaded polymeric nanoparticles (ANPs) before considering their application as a drug carrier via the oral route.

**Methods:** We synthesized N-isopropylacrylamide, N-vinyl pyrrolidone, and acrylic acid crosslinked polymeric NPs by free-radical polymerization reaction and characterized them by Fourier-transform infrared spectroscopy, transmission electron microscopy, and dynamic light scattering spectroscopy. Different concentrations of extract (50 mg/kg, 300 mg/kg, and 2,000 mg/kg body weight) were encapsulated into the hydrophobic core of polymeric micelles for the assessment of acute oral toxicity and their LD50 cut-off value as per the test procedure of OECD guideline 423. Orally administered female Wistar rats were observed for general appearance, behavioral changes, and mortality for the first 30 min, 4 h, 24 h, and then, daily once for 14 days.

**Result:** ANPs at the dose of 300 mg/kg body weight were used as an initial dose, and rats showed few short-lived signs of toxicity, with few histological alterations in the kidney and intestine. Based on these observations, the next set of rats were treated at a lower dose of 50 mg/kg and a higher dose of 2,000 mg/kg ANPs. Rats administered with 50 mg/kg ANPs remained normal throughout the study with insignificant histological disintegration; however, rats treated at 2,000 mg/kg ANPs showed some signs of toxicity followed by mortality among all three rats within 24–36 h, affecting the intestine, liver, and kidney. There were no significant differences in hematological and biochemical parameters among rats treated at 50 mg/kg and 300 mg/kg ANPs.

**Conclusion:** We conclude that the LD50 cut-off value of these ANPs will be 500 mg/kg extract loaded in polymeric NPs.

## 1 Introduction

*Artemisia absinthium* L. (Wormwood), a member of the family Asteraceae, is a perennial herb that is found mainly in the regions of Eurasia, North and South America, and Northern Africa (Bora and Sharma, 2010). This plant is known for its prominent ethnopharmacological significance and is used as a remedy in many diseases due to its antibacterial, antihelminthic, antifungal, antiparasitic, antiseptic, anti-inflammatory, and anticancer activities (Bhat et al., 2018; Mughees et al., 2019). All these activities are ascribed to the presence of different flavonoids, phenolics, and tannins whose type varies with varying geographical origin. The chief components of *A. absinthium* essential oil are β-thujone, β-pinene, (Z)-epoxyocimene, borneol, chrysanthenyl acetate, isobornyl acetate, and methyl hinokiate (Riahi et al., 2015). Oleic and linoleic saturated acids (palmitic and stearic), as well as epoxy oleic acid (oxirane), constitute the fatty acid portion of the essential oil. Phenolic compounds of the plant include p-hydroxyphenylacetic, protocatechuic, fisetin, isorhamnetin, and kaempferol, while phenolic acids include syringic, gallic, p-coumaric, vanillic, caffeic, ferulic, and chlorogenic acids, which are accountable for the antioxidant and free-radical scavenging potential of the plant (Craciunescu et al., 2012; Goud et al., 2015). Flavonoids include artemitin, quercetin, luteolin, apigenin, rutin, myricetin, spinacetin, and glycosides of quercetin, e.g., quercitin 3-glucoside, quercitin 3-rhamnoglucoside, spinacetin 3-glucoside, and spinacetin 3-rhamnoglucoside. Lignans (derived from phenylalanine) are polyphenolic compounds that include diayangambin and epiyangambin (Bora and Sharma, 2010). Volatile oils majorly contain sabinene, myrcene, linalool, and trans-sabinyl acetate. Monoterpene hydrocarbons are the principal component present in the plant with fewer oxygenated monoterpenes, sesquiterpene hydrocarbons, and oxygenated sesquiterpenes. Sesquiterpenes including α-bisabolol, β-curcumene, matricin, and spathulenol are mainly responsible for the antibacterial and antiparasitic activities of the plant (Goud et al., 2015).

Nanotechnology in the field of pharmaceuticals has been implemented to assist site-specific drug delivery by utilizing nanoparticles as a drug carrier which ensures efficient solubility, absorption, and bioavailability of several drugs. Different types of nanoparticles (NPs) are synthesized that vary in their morphology, size, and physical and chemical characteristics, e.g., lipid-based, metallic, ceramic, semiconductor, and polymeric nanoparticles. Among these, polymeric NPs are often preferred over other NPs as they possess good retention time, simple preparation, and less toxicity. Drug-loaded polymeric NPs can pass through the physiological barriers and further stimuli-triggered release of the desired concentration of drug into the intracellular compartment of the target site, thus making it ideal for the drug delivery system (Sur et al., 2019). Self-assembled, double-triggered thermoresponsive, and pH-sensitive crosslinked copolymeric micelles, made of N-isopropyl acrylamide (NIPAAM), N-vinyl pyrrolidone (VP), and acrylic acid (AA), are non-toxic and biocompatible and have been used previously with several drugs (e.g., ketorolac and riluzole) to ensure site-specific delivery of the formulation (Gupta et al., 2000; Verma et al., 2016). Copolymerization of the acrylic acid provides a carboxylic group, which makes it pH-sensitive and responsive when the pH is changed at the particular cell or tissue in the body. Similarly, NIPAAM, being a thermoresponsive polymer, has been known to have lower critical solution temperature (LCST) (32°C) near physiological temperature, and its copolymerization increases its LCST above which its polymeric form collapsed. The anticancer therapeutic potential of *A. absinthium* ethanolic extract-loaded NIPAAM–VP–AA NPs against breast cancer cell lines (MCF-7 and MDA MB-231) was previously evaluated by our research team (Mughees et al., 2020). The tumor microenvironment (leaky vasculature with irregular epithelial cells, elevated temperature around cancerous cells due to inflammation, and low physiological pH due to an acidic environment) was utilized for the targeted drug delivery system employing pH and thermoresponsive polymeric nanoparticles for *in vitro* breast cancer treatment.

The *A. absinthium* extract in conjugation with NIPAAM–VP–AA NPs would aid the establishment of potential drug with enhanced bioavailability, retention time, and efficacy of the extract due to site-specific drug delivery. The *in vivo* behavior of these NPs and their capability to cross certain biological barriers after oral administration have not been scrutinized previously. The oral utilization of nanoformulations with a larger surface-area-to-volume ratio provides greater surface area for mucosal interaction and adherence, protecting entrapped therapeutic agents from extreme pH conditions and enzymatic degradation and facilitating sustained release and enhanced systemic absorption of drugs or intact NPs in the gastrointestinal (GI) tract. However, the fate of NPs in the GI tract and further distribution throughout the body depends on their size, shape, composition, surface charge, conformation, and concentration (Peppas and Kavimandan, 2006; Hua, 2020). As inadequate information is available in the scientific literature about *in vivo* oral administration of the *A. absinthium* ethanolic extract and NIPAAM–VP–AA polymeric NPs, thus, the investigation of the *in vivo* safety of *A. absinthium* ethanolic extract-loaded NIPAAM–VP–AA NPs (ANPs) is a prerequisite before considering their clinical implication as a drug carrier via the oral route.

The main objectives of the present study were to identify the composition of the ethanolic extract of *A. absinthium* grown in the Indian subcontinent and the evaluation of the acute oral toxicity of these *A. absinthium* extract-loaded polymeric nanoparticles in rats as per the OECD guidelines for the Testing of Chemicals (423), Acute Oral Toxicity—Acute Toxic Class Method. This guideline will also allow the determination of the LD50 cut-off value of these ANPs and their further ranking for classification purposes and hazard assessment.

## 2 Materials and methods

### 2.1 Reagents

N-isopropyl acrylamide (NIPAAM) was procured from Sigma-Aldrich (United States) and recrystallized with N-hexane before use. N-vinyl pyrrolidone (VP) was purchased from Acros Organics, which was freshly distilled before use. Acrylic acid (AA) and N, N′-methylenebisacrylamide (MBA) were procured from Sigma-Aldrich (United States). Ferrous ammonium sulfate (FAS) and ammonium persulfate (APS) were purchased from SRL Pvt. Ltd. and Hi-media. Absolute ethanol was purchased from Merck (Germany).

### 2.2 Plant material and preparation of the ethanolic extract

Plant *A. absinthium* (vegetative stage) was obtained during mid-summer from Jamia Hamdard Herbal Garden. It was thoroughly washed to remove dust or dirt, and its different parts (root, leaf, and aerial portion) were shade-dried. Whole plant extract synthesis was carried out through maceration of 5 gm dried powdered sample mixed with 20 mL ethanol (1:5 = drug:solvent ratio) at 28°C ± 2°C for 24 h in an incubator shaker. This procedure was repeated three times consecutively, and the final collected solvent was filtered through Whatman filter paper no. 45. A rotary evaporator was used to concentrate the filtrate under vacuum pressure at 40°C until 5 mL solvent was left (with extraction yield = 3.06%), which was further filtered through a 0.22 µm Millipore syringe filter and stored at 4°C for further use.

### 2.3 Gas chromatography–mass spectrometry analysis

The types of active compounds present in the whole plant ethanolic extract of *A. absinthium* grown in the Indian subcontinent have not been reported previously. Therefore, it needs to be addressed before their therapeutic application. Thus, the separation and identification of compounds in the *A. absinthium* ethanolic extract were performed using a GC/MS QP 2010 (Shimadzu) equipped with an auto-injector (AOC-20i + s) mass selective detector having an ion source temperature of 220°C, interface temperature of 270°C, solvent cut time of 3.50 min, threshold of 1,000 eV, and mass range of 40–650 m/z. Compounds were separated using an Rxi-5 Sil MS capillary column (Restek Company, Bellefonte, United States) having dimensions 30 m × 0.25 mm × 0.25 μm (film thickness). The split injection mode was used at a split ratio of 10:1 having an injection temperature of 260°C. 1.0 μL of ethanolic extract was fixed as the injected volume. Oven temperature program started from 80°C (3 min) and further increased to 300°C at a rate of 10°C/min (16 min hold). Helium was used as the carrier gas at a linear flow velocity of 40.5 cm/s with a total and column flow fixed at 16.3 and 1.21 mL/min, respectively. Compounds were identified by the comparison of their relative retention time and mass spectra with those in the NIST libraries and literature data.

### 2.4 Synthesis and characterization of polymeric nanoparticles

Polymeric nanoparticles were synthesized as described previously (Mughees et al., 2020). Monomeric NIPAAM, VP, and AA were used in the molar ratio of 90:10:5. This protocol is based on the free-radical mechanism where MBA was used for crosslinking the monomers, FAS was used as an activator, and APS as an initiator in the polymerization reaction. Then, 180 mg NIPAAM, 20 µL VP, and 10 µL AA were dissolved in 20 mL double distilled water with vigorous vortexing. For crosslinking the monomers, 100 µL MBA (0.049 g/mL) was added and nitrogen gas was passed for 1 h to remove dissolved oxygen from the reaction. Then, 60 µL FAS (5 mg/mL) and 100 µL APS (saturated) were added in the reaction to trigger polymerization that lasted for 24 h at 32°C under nitrogen atmosphere. After completion, the final solution was dialyzed through a cellulose dialyzing membrane (cut-off 12 kDa) and lyophilized for further use.

Synthesized NPs were characterized for their average size distribution and polydispersity index (PDI) by Zetasizer Nano ZS Ver. 7.13, (Malvern Instruments Ltd., Worcestershire, United Kingdom) through dynamic scattering light (DLS) spectroscopy. 2 mL aqueous solution was used for measurement employing a laser beam (wavelength of 633 nm) passing through the solution at 25°C with a detection angle of 90°.

Transmission electron microscopy (TEM) was performed on Tecnai G20 HR-TEM (Thermo Scientific) operated at a voltage of 200 kV to analyze the size and morphology of synthesized NPs. A drop of NPs was placed on a carbon-coated copper grid, and 2% uranyl acetate was added. The grid was air-dried, and TEM images were obtained through a high-resolution digital CCD camera with image processing software (Olympus Soft Imaging System, Germany).

Fourier-transform infrared (FTIR) spectra of NIPAAM, VP, and AA and synthesized NPs were recorded through a Varian 7000 FTIR Spectrometer with a Varian 600 UMA Microscope using the KBr pellet method. Each monomer and NPs were scanned at wavenumbers in the range of 4,000 cm^−1^ to 400 cm^−1^ with a resolution of 2 cm^−1^ and 16 scans per sample.

### 2.5 Animals

Twenty-one 8–12-week-old (100–150 gm) healthy female Wistar rats were procured from the Central Animal House Facility (CAHF), Jamia Hamdard, New Delhi, and randomly housed in a group of three per cage under standard conditions (20 ± 2°C; 50 ± 10% relative humidity; and 12 h of light/dark cycles) for acclimatization to ideal laboratory conditions 7 days before the start of the experiment. The rats were provided free access to food and water *ad libitum*. All the experiments were approved and performed in compliance with the Institutional Animal Ethics Committee (IAEC) constituted through the Committee for the Purpose of Control and Supervision of Experiments on Animals (173/GO/Re/S/2000/CPCSEA) under the Ministry of Animal Welfare Division, Government of India, New Delhi.

### 2.6 Acute oral toxicity study

The toxicological assessment was conducted according to the procedures of the Organization for Economic Co-operation and Development (OECD) guideline for the Testing of Chemicals (423), Acute Oral Toxicity—Acute Toxic Class Method, which is a (single dose) 14-day acute oral study using three female Wistar rats per step. The range of acute toxicity of the test substance depends on the mortality incidence of the animals which assists in finding the toxicity category of the tested drug defined by fixed LD50 cut-off values. For animal welfare reasons, the OECD guideline 423 recommends using 300 mg/kg body weight as a starting dose, when sufficient information is not available about the substance to be tested. Lyophilized NPs were dissolved in distilled water, and the extract was added slowly with continuous vortexing and mild sonication to facilitate its physical entrapment inside the hydrophobic core of NPs. Different concentrations of *A. absinthium* ethanolic extract were loaded into NPs as per the body weight of each rat, and food but not water was withheld overnight before dosing. Group CN (n = 6) served as the control that received distilled water via oral gavage, while group ANP_300_ (n = 6) received *A. absinthium* ethanolic extract-loaded NPs at an initial dose of 300 mg/kg body weight. The rats were critically observed for general appearance, behavioral changes, and mortality rate for the first 30 min, 4 h, 24 h, and then, daily once for 14 days. The body weight of each rat was monitored and recorded periodically throughout the study.

Based on the absence or presence of mortality, group ANP _2000_ (n = 3) received the next higher dose of 2,000 mg/kg body weight ANPs. With a minor modification to the OECD guideline 423 due to the presence of few toxic signs among ANP_300_-treated animals, another group ANP_50_ (n = 6) received the next lower dose of 50 mg/kg body weight ANPs. On the 15th day, blood was drawn from the tail vein in EDTA-coated sterilized vials and anticoagulant free vials for whole blood and serum collection, respectively. The rats were euthanized by CO_2_ inhalation for procuring all the vital organs (stomach, intestine liver, kidney, spleen, heart, and brain) for necropsy, macroscopic examination, and histopathological analysis.

### 2.7 Hematological and biochemical parameters

Whole blood was processed immediately to examine hematological parameters using an automatic hematological analyzer (Sysmex XP 100, Transasia, India). The hematological parameters measured were hemoglobin (Hb), total leucocyte count (TLC), neutrophil, lymphocyte, eosinophil, monocyte, basophil, and red blood cell count (RBC), hematocrit (PCV/HCT), mean corpuscular volume (MCV), mean corpuscular Hb (MCH), mean corpuscular Hb concentration (MCHC), and platelet count. Anticoagulant-free vials were left undisturbed for 30 min to coagulate and then centrifuged for 15 min at 4,500 rpm for serum separation. Serum was used to investigate biochemical parameters using an automatic chemistry analyzer (ERBACHEM 7X, Transasia, India) comprising total bilirubin, direct bilirubin, indirect bilirubin, serum glutamic-oxaloacetic transaminase (SGOT), serum glutamic pyruvic transaminase (SGPT), alkaline phosphatase, total protein, albumin, globulin, urea, creatinine, uric acid, serum calcium, sodium, potassium, and chloride.

### 2.8 Histopathology

Various vital organs (stomach, intestine liver, kidney, spleen, heart, and brain) were fixed in a 10% buffered formalin solution before embedding in paraffin wax. From these blocks, 3–4 μm sections were made and stained with hematoxylin and eosin (H&E). Histopathology was carried out to identify any morphological and degenerative changes in different organs due to ANP administration.

### 2.9 Statistical analysis

The data obtained from all the groups were statistically analyzed by GraphPad Prism Version 7 and expressed as the mean ± standard deviation. Differences in hematology and biochemistry data were analyzed using one-way ANOVA followed by Dunnett’s multiple comparison tests. However, body weight results were analyzed using two-way ANOVA followed by Dunnett’s multiple comparison tests, and *p*-value < 0.05 was considered statistically significant.

## 3 Results

### 3.1 Gas chromatography–mass spectrometry analysis

**FIGURE 1:**
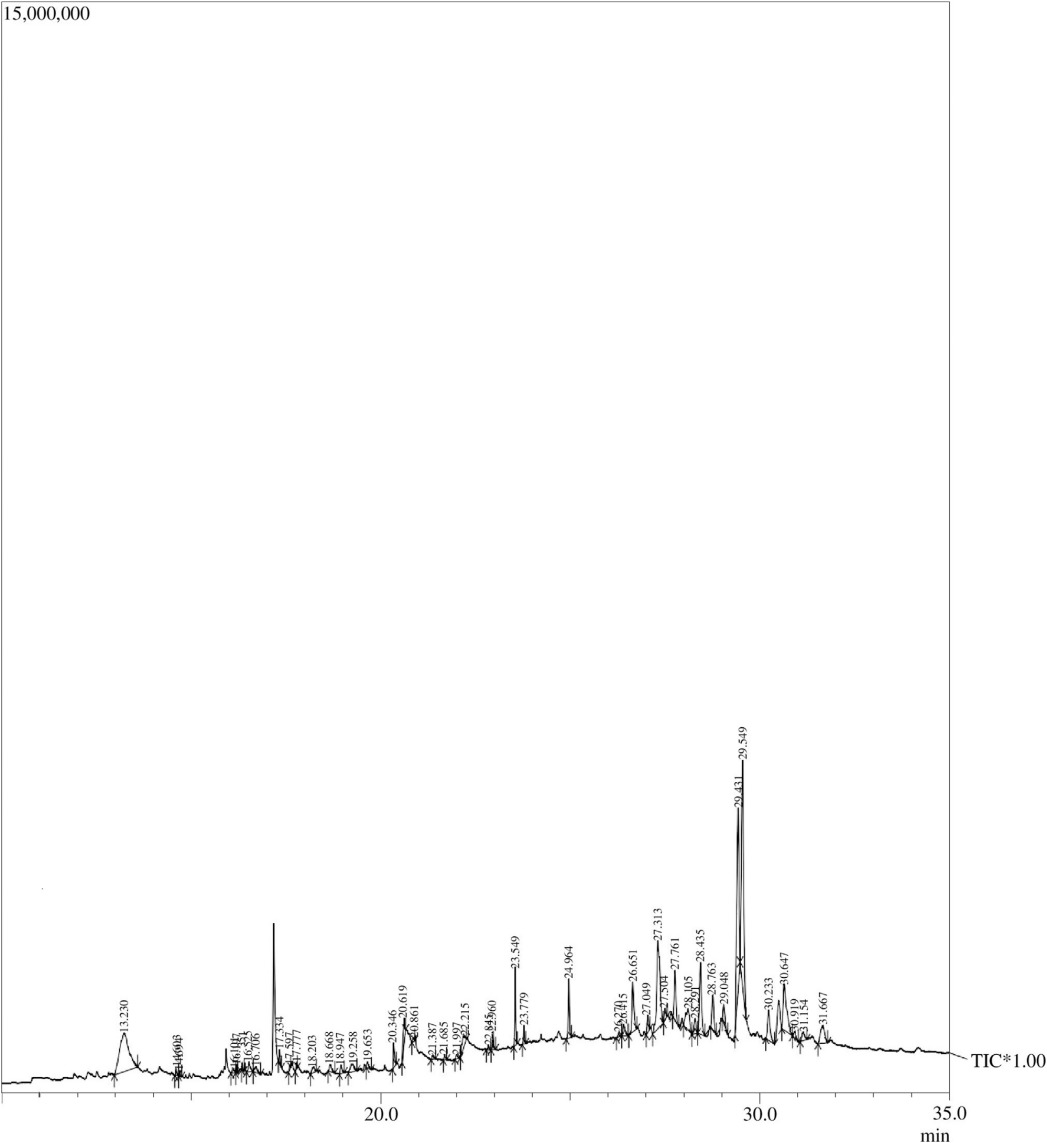
GC–MS chromatogram: Peaks of the *Artemisia absinthium* ethanolic extract obtained through GC–MS analysis.

Forty-five compounds were identified in the *A. absinthium* ethanolic extract through gas chromatography–mass spectrometry (GC–MS) analysis as shown in Figure 1 (chromatogram). The list of the active compounds, their retention time, molecular formula, and area % are shown in Table 1. All the detected compounds belong to diverse classes of phytochemicals with ethnomedical benefits, namely, polysaccharides, terpenoids, flavonoids, phytosterols, lignans, and fatty acid esters. Out of the forty-five compounds, some biologically important and prevalent compounds include mome inositol (11.83%), tetracontane (2.26%), stigmasta-5,22-dien-3-ol (3.34%), gamma-sitosterol (9.55%), cycloartenol (1.07%), yangambin (34.44%), 24-methylenecycloartanol (1.08%), and sesartemin (5.28%), while compounds like neophytadiene (0.25%), 6,10,14-trimethyl-2-pentadecanone (0.16%), hexadecanoic acid, ethyl ester (0.23%), alpha-curcumene (0.24%), reynosin (0.54%), phytol (0.48%), 9,12-octadecadienoic acid (Z,Z)-, methyl ester (0.25%), isogeraniol (0.47%), 1-docosanol (0.48%), 2-methyloctacosane (0.23%), squalene (0.67%), ergost-5-en-3-ol, (3Beta,24R) (0.87%), and artemetin (0.61%) were present in trace amount as shown in Table 2. Phytosterols, including gamma-sitosterol (Sundarraj et al., 2012), campesterol, and stigmasta-5,22-dien-3-ol (Shahzad et al., 2017; Vezza et al., 2020), have the potential to trigger the production of anti-inflammatory cytokine, hepatoprotective, hypercholesterolemic, antidiabetic, and anticancer activities. The two most dominant lignans, namely, yangambin (Monte et al., 2007; Bala et al., 2015) and sesartemin (Dixit and Reddy, 2017; Ickovski et al., 2020), tend to exhibit analgesic, antianaphylactic, antiallergic, antileishmanial, anti-PAF, and anticancer activities and can act as an inhibitor of cytochrome P450-linked oxygenase. Polysaccharide-like mome inositol has been reported with antiallopathic, anticirrhotic, antineuropathic, cholesterolytic, and lipotropic activities (Das et al., 2014). Flavonoids like artemetin possess antioxidant, hepatoprotective, and antiedematogenic activities (Bayeux et al., 2002). Palmitic and linoleic acid esters, namely, hexadecanoic acid, ethyl ester, and 9,12-octadecadienoic acid (Z, Z)-, methyl ester, also have antioxidant, anti-inflammatory, hypocholesterolemic, cancer preventive, insectifuge, antiarthritic, hepatoprotective, antiandrogenic, nematicide, and antihistaminic properties (Krishnamoorthy and Subramaniam, 2014). Compounds belonging to different classes of terpenes, such as neophytadiene (Bhardwaj et al., 2020), 6,10,14-trimethyl-2-pentadecanone (Chen et al., 2022), alpha-curcumene (Obrenovich et al., 2010), reynosin (Lim et al., 2013), phytol (Krishnamoorthy and Subramaniam, 2014), isogeraniol (Lei et al., 2019), squalene, cycloartenol, and 24-methylenecycloartenol (Wu et al., 2022), have been found to encompass antioxidant, anti-inflammatory, antimicrobial, antiproliferative, immunostimulant, chemopreventive, lipoxygenase-inhibitor, pesticide, and diuretic activities. Polycosanols including 1-docosanol and tetracontane (Vergara et al., 2015) have anti-inflammatory, antiproliferative, analgesic, and antiviral activities, while isononacosane-like 2-methyloctacosane has an antimicrobial activity (Barretto and Vootla, 2018).

**TABLE 1:** Compounds identified in *Artemisia absinthium* whole plant ethanolic extract through GC–MS analysis.

Peak	R.Time	Area	Name	Formula	Area%
1	13.230	8,993,640	Mome inositol	C_7_H_14_O_6_	11.83
2	14.603	190,918	Neophytadiene	C_20_H_38_	0.25
3	14.694	119,615	6,10,14-Trimethyl-2-pentadecanone	C_18_H_36_O	0.16
4	16.101	205,452	4-tert-Butyl-2,6-diisopropylphenol	C_16_H_26_O	0.27
5	16.197	178,382	Hexadecanoic acid, ethyl ester	C_18_H_36_O_2_	0.23
6	16.351	179,208	alpha-Curcumene	C_15_H_22_	0.24
7	16.525	488,604	Cyclohexanemethanol, 4-ethenyl-alpha.,.Alpha.,4-Trimethyl-3-(1-methylethenyl)-, [1R (1.Alpha.,3.Alpha.,4.Beta.)]	C_15_H_26_O	0.64
8	16.706	408,294	Reynosin	C_15_H_20_O_3_	0.54
9	17.334	363,358	Phytol	C_20_H_40_O	0.48
10	17.597	121,731	3,5-Dimethylcyclohex-1-ene-4-carboxaldehyde	C_9_H_14_O	0.16
11	17.777	189,641	9,12-Octadecadienoic acid (Z,Z)-, methyl ester	C_19_H_34_O_2_	0.25
12	18.203	368,050	11-Heneicosanone	C_21_H_42_O	0.48
13	18.668	359,300	Isogeraniol	C_10_H_18_O	0.47
14	18.947	362,183	1-Docosanol	C_22_H_46_O	0.48
15	19.258	718,469	13,21-Cyclo-18-norpregnan-20-ol, 20-methyl-, (5.alpha.,20S	C_21_H_34_O	0.95
16	19.653	294,295	p-Undecyloxybenzoic acid	C_18_H_28_O3	0.39
17	20.346	934,622	Silane, dimethyl(3-ethylphenoxy)heptyloxy-	C_17_H_30_O_2_Si	1.23
18	20.619	1,168,896	1-Heneicosanol	C_21_H_44_O	1.54
19	20.861	129,421	Palmitic acid, TMS derivative	C_19_H_40_O_2_Si	0.17
20	21.387	174,184	2-Methyloctacosane	C_29_H_60_	0.23
21	21.685	168,280	(E)-3,7-dimethylocta-2,6-dien-1-yl stearate	C_28_H_52_O_2_	0.22
22	21.997	202,569	1H-indole-3-acetic acid	C_10_H_9_NO_2_	0.27
23	22.215	982,203	Hexanoic acid, heptadecyl ester	C_23_H_46_O_2_	1.29
24	22.845	172,657	Pentacontanoic acid, ethyl ester	C_52_H_104_O_2_	0.23
25	22.960	507,808	Squalene	C_30_H_50_	0.67
26	23.549	1,766,666	Pentacosane	C_25_H_52_	2.32
27	23.779	445,163	2,2-Dimethyl-3-(3,7,16,20-tetramethyl-heneicosa-3,7,11,15, 19-pentaenyl)-oxirane	C_29_H_48_O	0.59
28	24.964	1,715,947	Tetracontane	C_40_H_82_	2.26
29	26.270	177,153	2,4-Diamino-5-[3,4,5-trimethoxybenzoxy]pyrimidine	C_14_H_16_N_4_O_5_	0.23
30	26.415	664,124	Ergost-5-en-3-ol, (3Beta,24R)-	C_28_H_48_O	0.87
31	26.651	2,537,184	Stigmasta-5,22-dien-3-ol	C_29_H_48_O	3.34
32	27.049	643,954	Ergosta-8,24(28)-dien-3-Ol, 4,14-dimethyl-, (3.Beta.,4.Alpha.,5.Alpha.)	C_30_H_50_O	0.85
33	27.313	7,256,424	gamma-Sitosterol	C_29_H_50_O	9.55
34	27.504	459,989	4H-1-benzopyran-4-one, 2-(3,4-dimethoxyphenyl)-5-hydroxy-3,6,7-trimethoxy; artemetin	C_20_H_20_O_8_	0.61
35	27.761	2,265,533	3-Debenzoyl-tetrahydrocarpesterol	C_30_H_54_O_3_	2.98
36	28.105	2,022,248	5-Chloro-3-(4-methoxyphenyl)-1-methylpyrazole-4-carboxaldehyde	C_12_H_11_ClN_2_O_2_	2.66
37	28.291	812,278	9,19-Cyclolanost-24-en-3-ol, (3.beta.)-; cycloartenol	C_30_H_50_O	1.07
38	87.415	26,175,102	Yangambin	C_24_H_30_O_8_	34.44
39	28.763	1,641,286	Cholesterol epoxide	C_27_H_46_O_2_	2.16
40	29.048	822,805	24-Methylenecycloartenol	C_31_H_52_O	1.08
41	30.233	1,870,633	1-Meyhyl-2,4,6-triphenyl-1-[2-phenyl-(E)-ethenyl]-.Lambda(5).-Phosphinine	C_32_H_27_P	2.46
42	30.647	4,011,010	Sesartemin	C_23_H_26_O_8_	5.28
43	30.919	349,032	3-(1,5-Dimethyl-hexyl)-3a,10,10,12b-tetramethyl-1,2,3,3a,4,6,8,9,10,10a,11,12,12a,12b-tetradecahydro-benzo[4,5]cyclohepta[1,2-E]inde ne	C_30_H_50_	0.46
44	31.154	551,541	Dimethyl 2-[(1′,4′-dimethoxy-9′,10′-dioxo-9′,10′dihydroanthracen-2′-Yl)Methylene]Butanedioate	C_23_H_22_O_8_	0.73
45	31.667	1,831,203	4.alpha.,14-dimethyl-5.alpha.-ergosta-8,24(28)-dien-3.beta-ol	C_30_H_50_O	2.41

**TABLE 2:** Activity of some important bioactive compounds found in *Artemisia absinthium* whole plant ethanolic extract.

Peak	Name	Compound nature	Activity
**1.**	Mome inositol	Polysaccharide	Anticirrhotic, antineuropathic, antioxidant, cholesterolytic, and lipotropic; Das et al. (2014)
**2.**	Neophytadiene	Diterpene	Anti-inflammatory and antimicrobial; Bhardwaj et al. (2020)
**3.**	6,10,14-Trimethyl-2-pentadecanone	Sesquiterpene	Anti-inflammatory and antibacterial; Chen et al. (2022)
**4.**	Hexadecanoic acid, ethyl ester	Palmitic acid	Antioxidant and anti-inflammatory; Krishnamoorthy and Subramaniam, (2014)
**5.**	alpha-Curcumene	Sesquiterpene	Antioxidant, anti-inflammatory, antimicrobial, cytotoxic, and antiviral; Obrenovich et al. (2010)
**6.**	Reynosin	Sesquiterpene	Hepatoprotective, anti-inflammatory, and antimicrobial; Lim et al. (2013)
**7.**	Phytol	Diterpene	Antimicrobial, anti-inflammatory anticancer, diuretic, antitumor, chemopreventive, and antimicrobial; Krishnamoorthy and Subramaniam, (2014)
**8.**	9,12-Octadecadienoic acid (Z, Z)-, methyl ester	Linoleic acid ester	Anti-inflammatory, hypocholesterolemic, cancer preventive, insectifuge, antiarthritic, hepatoprotective, antiandrogenic, nematicide, and antihistaminic; Krishnamoorthy and Subramaniam, (2014)
**9.**	Isogeraniol	Terpene	Flavoring agent and antifungal; Lei et al. (2019)
**10.**	1-Docosanol	Polycosanols	Antiproliferative and antiviral; Vergara et al. (2015)
**11.**	2-Methyloctacosane	Hydrocarbon	Antimicrobial; Barretto and Vootla, (2018)
**12.**	Squalene	Triterpene	Antibacterial, antioxidant, antitumor, cancer preventive, immunostimulant, chemopreventive, lipoxygenase-inhibitor, and pesticide; Wu et al. (2022)
**13.**	Tetracontane	Polycosanols	Anti-inflammatory and analgesic; Vergara et al. (2015)
**14.**	Ergost-5-en-3-ol, (3Beta,24R)-; campesterol	Phytosterol	Protection against liver diseases (e.g., jaundice), atherosclerosis, and anticancer; Shahzad et al. (2017); Vezza et al. (2020)
**15.**	Stigmasta-5,22-dien-3-ol	Phytosterol	Anti-inflammatory, antihypercholestrolemic, antitumor antioxidant, antiarthritic antiasthma, diuretic, antibacterial, and antiviral; Shahzad et al. (2017); Vezza et al. (2020)
**16.**	gamma-Sitosterol	Phytosterols	Antimicrobial, antidiabetic, and anticancer; Sundarraj et al. (2012)
**17.**	4H-1-benzopyran-4-one, 2-(3,4-dimethoxyphenyl)-5-hydroxy-3,6,7-trimethoxy; artemetin	Flavonoids	Antioxidant, hepatoprotective, and antiedematogenic; Bayeux et al. (2002)
**18.**	9,19-Cyclolanost-24-en-3-ol, (3.beta.)-; cycloartenol	Triterpenoid	Antioxidant and antimicrobial; Wu et al. (2022)
**19.**	Yangambin	Lignan	Analgesic, antianaphylactic, antiallergic, antileishmanial, anti-PAF, and anticancer; Monte et al. (2007); Bala et al. (2015)
**20.**	24-Methylenecycloartenol	Triterpenoid	Antiproliferative, arousal-effect, and anti-inflammatory; Wu et al. (2022)
**21.**	Sesartemin	Lignin	Inhibitor of cytochrome P450-linked oxygenase and cytotoxic; Dixit and Reddy, (2017); Ickovski et al. (2020)

### 3.2 Synthesis and characterization of the synthesized polymeric nanoparticles

**FIGURE 2:**
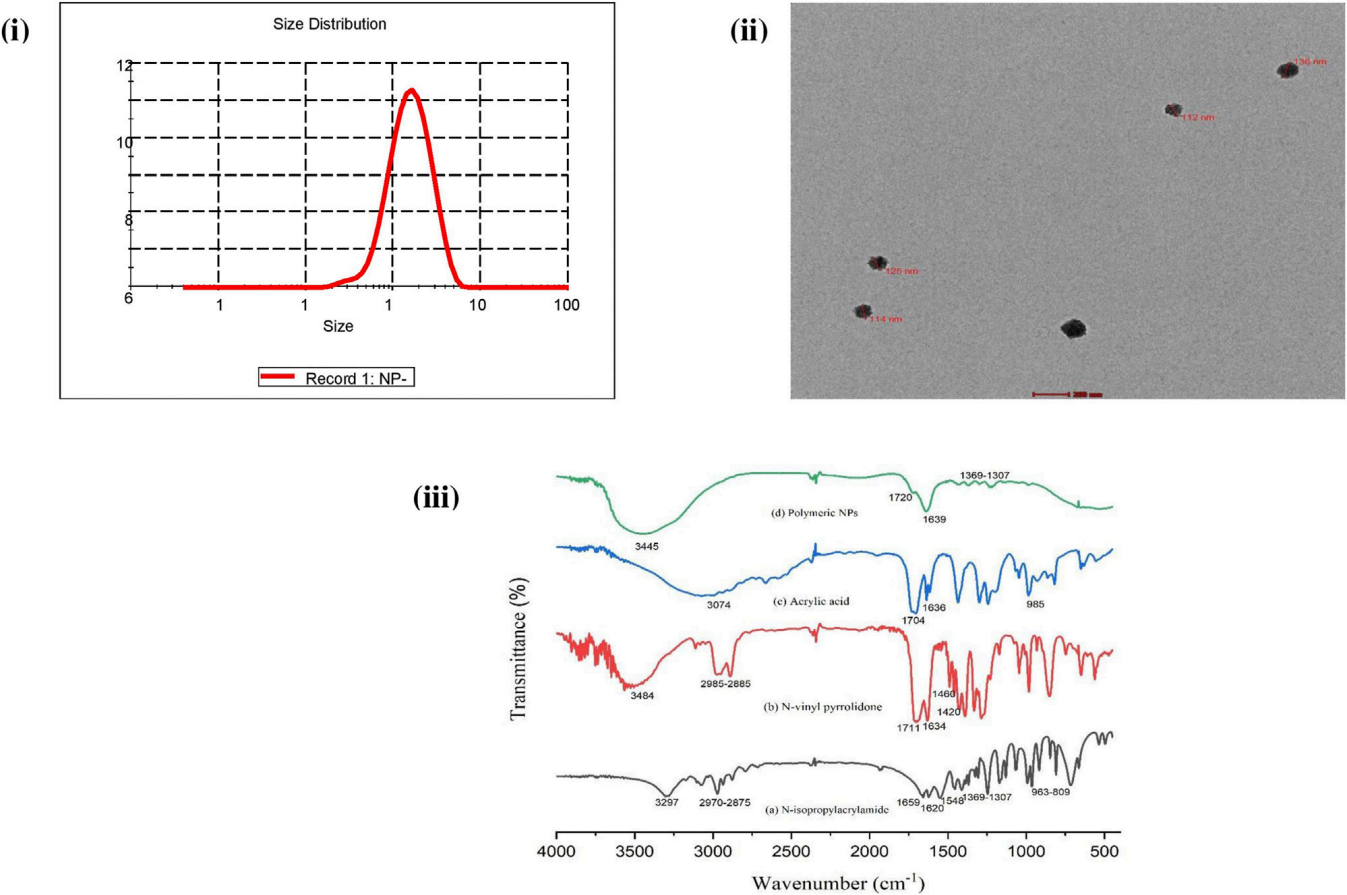
Characterization of synthesized NIPAAM–VP–AA polymeric nanoparticles: **(i)** DLS analysis showing the average size and PDI. **(ii)** TEM analysis showing the shape and size (scale bar = 0.2 μm). **(iii)** FT-IR spectroscopy analysis showing the IR spectra of (a) NIPAAM, (b) N-vinyl pyrrolidone (VP), (c) acrylic acid (AA), and (d) synthesized polymeric nanoparticles. Data are presented as the mean ± SD.

The free-radical polymerization process was exploited for the copolymerization of NIPAAM, VP, and AA monomers resulting in the formation of amphiphilic micelles with an external hydrophilic shell and an inner hydrophobic core. This hydrophobic core of the micelles was utilized as the carrier of the plant extract. The average particle size of NPs was found to be 131 nm with PDI 0.2 at 25°C as determined by DLS, indicating monodispersity in solution [Figure 2I]. TEM analysis revealed prominent spherical-shaped morphology of NPs with an average size of 117 nm ± 4.04 nm as shown in Figure 2II. FTIR spectra of monomeric NIPAAM, VP, AA, and synthesized polymeric NPs are shown in Figure 2III. FTIR spectra of NIPAAM [Figure 2IIIa] show characteristic absorption peaks at 3,297 cm^−1^ corresponding to N–H stretching vibrations of the secondary amide group. Absorption peaks in the range of 2,970–2,875 cm^−1^ correspond to C–H bond stretching vibrations of methyl and isopropyl groups in NIPAAM. The absorption peak at 1,659 cm^−1^ and 1,548 cm^−1^ occurred due to amide I-associated C=O stretching vibrations and amide II-associated N–H bending with C–N stretching vibrations, respectively. A medium-intensity absorption peak at 1,620 cm^−1^ originated from the stretching vibrations of the C=C bond, while peaks in the range of 963–809 cm^−1^ appeared due to the out-of-plane bending vibrations of = C–H bond in the vinyl group of NIPAAM. Other less-intense peaks in the range of 1,369–1,307 cm^−1^ correspond to vibrations from the isopropyl group of NIPAAM. FTIR spectra of VP [Figure 2IIIb] had characteristic absorption peaks at 3,484 cm^-1^ and 1,711 cm^−1^ occurring due to O–H and amide C=O stretching vibrations, respectively. Absorption peaks in the range 2,985–2,885 cm^−1^ and 1,634 cm^−1^ correspond to C–H and C=C stretching vibrations, respectively. Peaks at 1,420 cm^−1^ and 1,460 cm^−1^ occurred due to = C–H and aromatic ring stretching vibrations. FTIR spectra of AA [Figure 2IIIc] represent characteristic absorption peaks at 3,074 cm^−1^ and 1,704 cm^−1^ due to carbonyl O–H bond and C=O bond stretching vibrations, respectively. Additionally, absorption peaks at 1,636 cm^−1^ and 985 cm^−1^ are designated to C=C and = C–H stretching vibrations, respectively. However, FTIR spectra of synthesized polymeric NPs [Figure 2IIId] depict changes in the peaks corresponding to specific functional groups in monomers indicating the formation of new interactions. There were no peaks in the range of 800–1,000 cm^−1^ corresponding to stretching vibrations of vinyl double bond indicating that polymerization has occurred among monomers via breaking of the C=C bond (Larrañaga et al., 2011; Verma et al., 2016). Absorption peaks at 1,639 cm^−1^ and 1,720 cm^−1^ correspond to C=O stretching vibrations from all three monomeric units in the polymer. Another broad and intense peak at 3,445 cm^−1^ represents O–H stretching vibration due to attached water of hydration with the polymer (Gupta et al., 2000; Mughees et al., 2020). In comparison with the monomer, declination in the absorption peaks in the range of 1,369–1,307 cm^−1^ corresponding to vibrations from the isopropyl group implies their involvement as the crosslinking points.

### 3.3 Acute oral toxicity study

All rats were observed individually for several clinical signs of toxicity including loss of appetite, skin and fur change, sitting at the corner, mortality, convulsion, drowsiness, salivation, respiratory depression/irregular respiratory pattern, lacrimation, abnormal sleeping pattern, tremors, diarrhea, aggressiveness, and piloerection and recorded as shown in Table 3. Group ANP_300_ treated at an initial dose of 300 mg/kg appeared less active and drowsy, sat at the corners of the cage with loss of appetite for the first 30 min of the oral administration compared to control, and then, appeared normal afterward. The presence of these few clinical signs but no mortality in animals treated at 300 mg/kg led us to proceed to the next higher dose of 2,000 mg/kg and the next lower dose of 50 mg/kg. Group ANP_2000_ showed few signs of toxicity in the first 30 min which included loss of appetite, sitting at the corner, and drowsiness. Group ANP_2000_ animals were excluded as major toxicity signs appeared after 4 h of dosing that included lacrimation, diarrhea, aggressiveness, respiratory depression, and abnormal sleeping pattern, and all three animals died within 24–36 h. Macroscopic examination of organs dissected from dead animals did not reveal any necrosis, hemorrhage, or changes in size, shape, and color. Group ANP_50_ remains active and normal without showing any adverse signs of toxicity. As per the principle of the OECD guideline 423 shown in “Annexure 2c,” for all three animals dying at 2,000 mg/kg dose within 24–48 h, while all the animals surviving at 300 mg/kg dose during the study, the LD50 cut-off value of ANPs will be 500 mg/kg body weight extract loaded in polymeric NPs.

**TABLE 3:** Clinical signs of toxicity in Group CN (control), ANP_50_ (50 mg/kg), ANP_300_ (300 mg/kg), and ANP_2000_ (2,000 mg/kg) during the acute oral toxicity study of *Artemisia absinthium* whole extract-loaded nanoparticles using the OECD guideline 423.

	30 minutes				4 hours				24 hours				7 days				14 days			
Signs	CN	ANP_50_	ANP_300_	ANP_2000_	CN	ANP_50_	ANP_300_	ANP_2000_	CN	ANP_50_	ANP_300_	ANP_2000_	CN	ANP_50_	ANP_300_	ANP_2000_	CN	ANP_50_	ANP_300_	ANP_2000_
**Loss of appetite**	**-**	**-**	**+**	**+**	**-**	**-**	**-**	**+**	**-**	**-**	**-**	**+**	**-**	**-**	**-**	N.A.	**-**	**-**	**-**	N.A.
**Skin and fur change**	**-**	**-**	**-**	**-**	**-**	**-**	**-**	**-**	**-**	**-**	**-**	**-**	**-**	**-**	**-**	N.A.	**-**	**-**	**-**	N.A.
**Sitting at the corner**	**-**	**-**	**+**	**+**	**-**	**-**	**-**	**+**	**-**	**-**	**-**	**+**	**-**	**-**	**-**	N.A.	**-**	**-**	**-**	N.A.
**Mortality**	**-**	**-**	**-**	**-**	**-**	**-**	**-**	**-**	**-**	**-**	**-**	3/3	**-**	**-**	**-**	N.A.	**-**	**-**	**-**	N.A.
**Convulsion**	**-**	**-**	**-**	**-**	**-**	**-**	**-**	**-**	**-**	**-**	**-**	**-**	**-**	**-**	**-**	N.A.	**-**	**-**	**-**	N.A.
**Drowsiness**	**-**	**-**	**+**	**+**	**-**	**-**	**-**	**+**	**-**	**-**	**-**	**+**	**-**	**-**	**-**	N.A.	**-**	**-**	**-**	N.A.
**Salivation**	**-**	**-**	**-**	**-**	**-**	**-**	**-**	**-**	**-**	**-**	**-**	**-**	**-**	**-**	**-**	N.A.	**-**	**-**	**-**	N.A.
**Respiratory depression**	**-**	**-**	**-**	**-**	**-**	**-**	**-**	**+**	**-**	**-**	**-**	**+**	**-**	**-**	**-**	N.A.	**-**	**-**	**-**	N.A.
**Lacrimation**	**-**	**-**	**-**	**-**	**-**	**-**	**-**	**+**	**-**	**-**	**-**	**+**	**-**	**-**	**-**	N.A.	**-**	**-**	**-**	N.A.
**Abnormal sleeping pattern**	**-**	**-**	**-**	**-**	**-**	**-**	**-**	**+**	**-**	**-**	**-**	**+**	**-**	**-**	**-**	N.A.	**-**	**-**	**-**	N.A.
**Tremors**	**-**	**-**	**-**	**-**	**-**	**-**	**-**	**-**	**-**	**-**	**-**	**-**	**-**	**-**	**-**	N.A.	**-**	**-**	**-**	N.A.
**Diarrhea**	**-**	**-**	**-**	**-**	**-**	**-**	**-**	**+**	**-**	**-**	**-**	**+**	**-**	**-**	**-**	N.A.	**-**	**-**	**-**	N.A.
**Aggressiveness**	**-**	**-**	**-**	**-**	**-**	**-**	**-**	**+**	**-**	**-**	**-**	**+**	**-**	**-**	**-**	N.A.	**-**	**-**	**-**	N.A.
**Piloerection**	**-**	**-**	**-**	**-**	**-**	**-**	**-**	**-**	**-**	**-**	**-**	**-**	**-**	**-**	**-**	N.A.	**-**	**-**	**-**	N.A.

(−): absence of sign, (+): presence of sign, (3/3): three out of three rats died within 24 h, (N.A.): not applicable as rats died.

**FIGURE 3:**
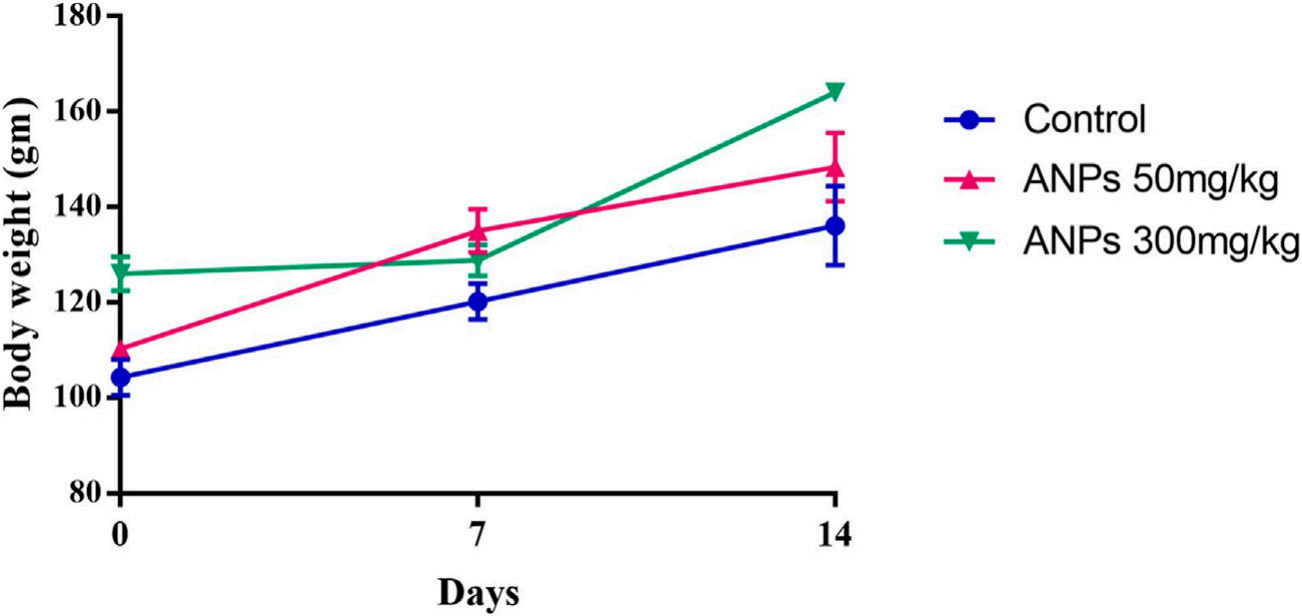
Increase in the mean value of body weight among control and treated groups. Significant increase in body weight was noted in control and groups treated at 50 mg/kg throughout 14 days. No significant growth was noticed in animals treated at 300 mg/kg till the 7th day, although weight gain resumed and continued till the 14th day. Values are expressed as the mean ± SD (N = 3); *p < 0.05 compared to the control.

A statistically significant increase in the mean value of body weight gain was observed in the control (*p* < 0.01), as well as group ANP_50_ treated at 50 mg/kg (*p* < 0.05) up to the 14th day as shown in Figure 3. However, the mean body weight of animals treated at 300 mg/kg ANPs remained quite stagnant, and no statistical change occurred till the seventh day, although weight gain resumed after 7 days and significantly increased till the 14th day (*p* < 0.001).

### 3.4 Histopathology

**FIGURE 4:**
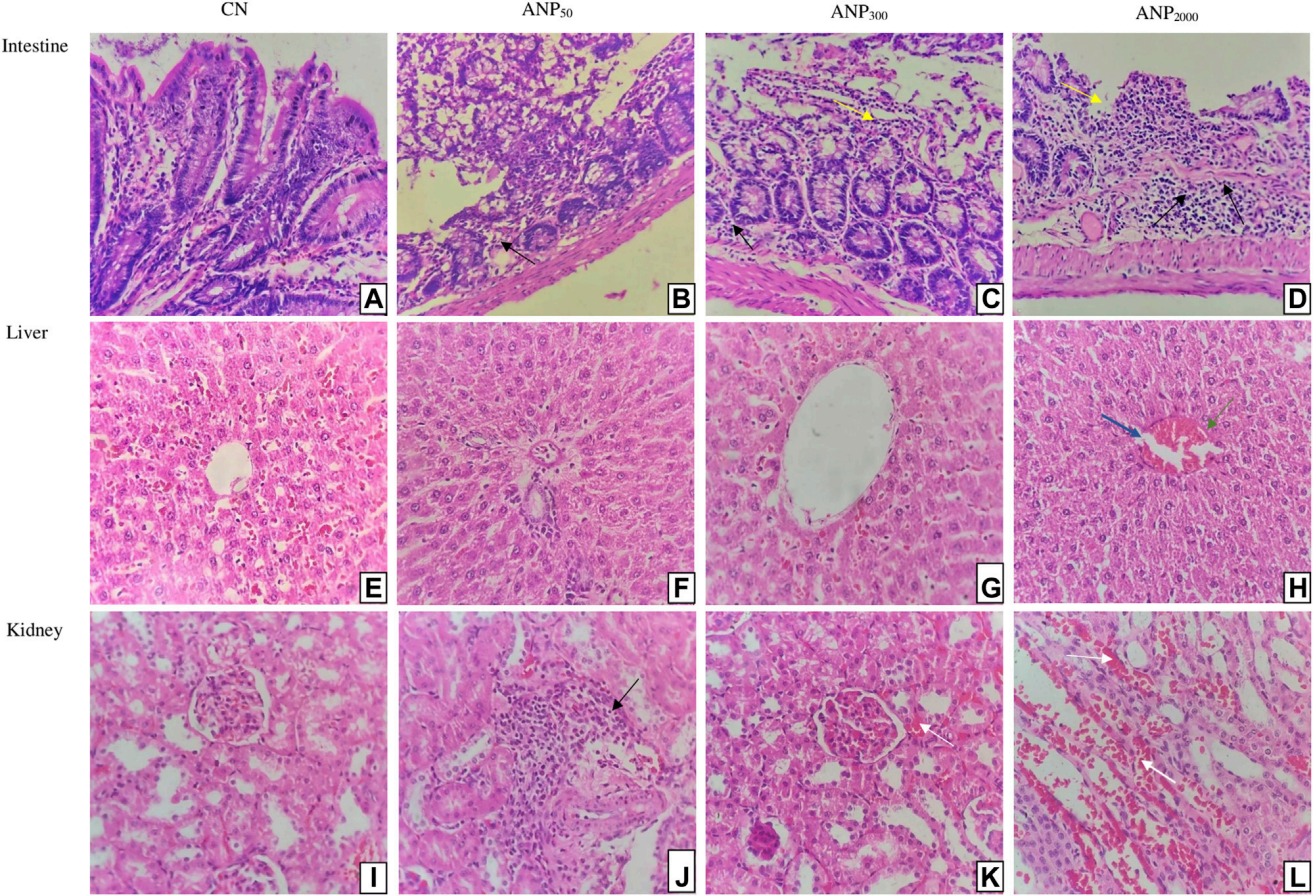
Histological sections of the intestine, liver, and kidney of rats (Hematoxylin and eosin stain, magnification 400×): Sections of Group **(A)** CN (control) and **(B)** ANP_50_ treated at 50 mg/kg show normal intestinal mucosa, submucosa, and muscularis propria; although ANP_50_ had insignificant inflammatory cell infiltrate; **(C)** ANP_300_ treated at 300 mg/kg show mild ulceration (Yellow arrow) with inflammatory infiltrates (Black arrows) in lamina propria; **(D)** ANP_2000_ treated at 2,000 mg/kg had ulceration (Yellow arrow) of intestinal mucosa with dense mixed inflammatory infiltrates (Black arrows) comprising of plasma cells, lymphocytes, and eosinophils in lamina propria (Yellow arrow depict ulceration of intestine and black arrows depict inflammatory infiltrates). Sections of Group **(E)** CN (control), **(F)** ANP_50_ treated at 50 mg/kg, **(G)** ANP_300_ treated at 300 mg/kg, and **(H)** ANP_2000_ treated at 2,000 mg/kg represents maintained lobular architecture, normal cell plate thickness, and polarity of hepatic parenchyma without necrosis, biliary cell damage or inflammatory infiltrate; although dilatation of sinusoids (Blue arrow) and few central veins with interspersed congested blood vessels (Green arrow) were also seen in all the groups (Blue arrow indicate dilated sinusoids and green arrow indicate congested central veins). Sections of Group **(I)** CN (control), **(J)** ANP_50_ treated at 50 mg/kg, **(K)** ANP_300_ treated at 300 mg/kg, and **(L)** ANP_2000_ treated at 2,000 mg/kg represent the kidney comprising of cortex and medulla where cortex shows numerous normal glomeruli having normal capillary loops and normal mesangial matrix deposition, normal proximal convoluted tubules, distal convoluted tubules, the loop of Henle and interstitium without any area of ischemia or necrosis; although **(J)** had focal areas of renal cortex with mild inflammatory infiltrate (Black arrow); **(K)** and **(L)** had focal areas of hemorrhage in the cortex (White arrow) (White arrow depict haemorrhage and black arrow depict inflammatory infiltrates).

Microscopic histopathological observations of the intestine, liver, and kidney of all the groups are shown in Figure 4. Figure 4A shows a normal intestinal architecture of group CN, and Figure 4B shows the normal intestinal architecture of group ANP_50_ with insignificant inflammatory cell infiltrate. Figure 4C shows minor ulceration and inflammatory cell infiltrate in the lamina propria, and Figure 4D shows ulceration of intestinal mucosa with dense mixed inflammatory infiltrates in the lamina propria of group ANP_300_ and ANP _2000_, respectively. Necropsy analysis of animals treated at 2,000 mg/kg ANPs reveals that a small amount of ANPs was deposited in the lumen of the intestine. These degenerative and corrosive effects of ANPs at intestinal pH can be attributed to the fact that ANPs were able to pass through the intestinal epithelium and reached the lamina propria. Representative photomicrographs of the liver [Figures 4E–G] from groups CN, ANP_50_, and ANP_300_ represent the preserved hepatic lobular architecture, normal cell plate thickness, and polarity of hepatic parenchyma without necrosis, biliary cell damage, or inflammatory infiltrate. However, dilatation of sinusoids and a few central veins with interspersed congested blood vessels without necrosis or inflammatory infiltrate were seen in all the groups which could be due to the impaired venous drainage at the time of death. Figures 4I–L belong to sections from the kidney comprising of the cortex and medulla of groups CN, ANP_50_, ANP_300_, and ANP_2000_ showing normal histological features of renal tubules and glomeruli with no evidence of ischemia or necrosis. However, Figure 4J of ANP_50_ shows focal areas of the cortex with inflammatory infiltrates. Figures 4K, L shows focal areas of hemorrhage in the renal cortex without any other architectural distortion in animals treated at 300 mg/kg and 2,000 mg/kg, respectively. After entering systemic circulation, the kidney could be the major site of action for these ANPs where they would have accumulated causing renal toxicity.

**FIGURE 5:**
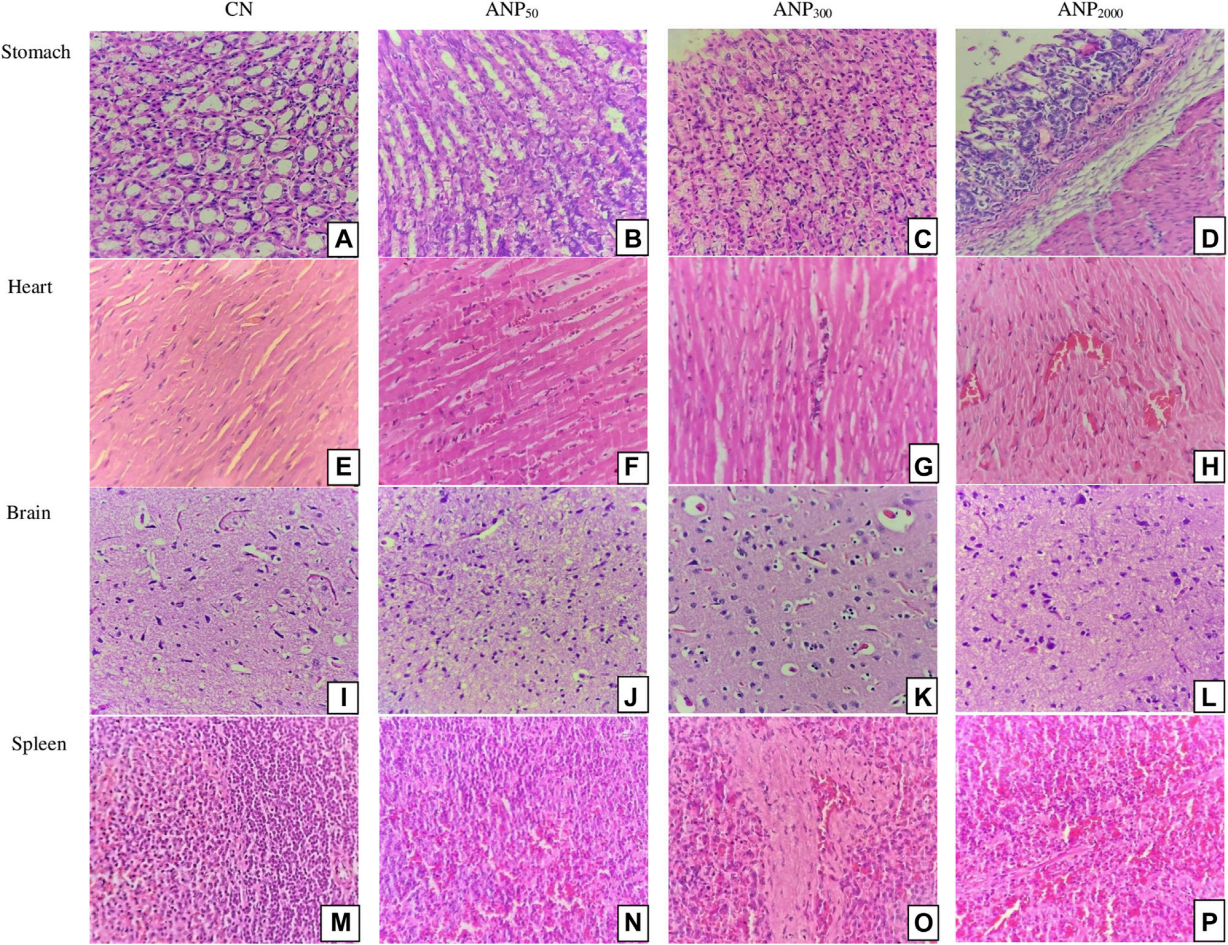
Histological sections of the stomach, heart, brain, and spleen of rats (hematoxylin and eosin stain, magnification ×400). Sections of group **(A)** CN (control), **(B)** ANP_50_ treated at 50 mg/kg, **(C)** ANP_300_ treated at 300 mg/kg, and **(D)** ANP_2000_ treated at 2,000 mg/kg show normal gastric mucosa comprising foveolar epithelium without any inflammation or necrosis. Micrographs of group **(E)** CN (control), **(F)** ANP_50_ treated at 50 mg/kg, **(G)** ANP_300_ treated at 300 mg/kg, and **(H)** ANP_2000_ treated at 2,000 mg/kg display normally arranged and maintained polarity of myocytes arranged in muscle bundles. Sections of group **(I)** CN (control), **(J)** ANP_50_ treated at 50 mg/kg **(K)** ANP_300_ treated at 300 mg/kg, and **(L)** ANP_2000_ treated at 2,000 mg/kg show normally arranged astrocytes, oligodendrocytes, axons, and interspersed capillaries in a fibrillary background. Micrographs of group **(M)** CN (control), **(N)** ANP_50_ treated at 50 mg/kg, **(O)** ANP_300_ treated at 300 mg/kg, and **(P)** ANP_2000_ treated at 2,000 mg/kg show normal white and red pulp of the spleen with a profused lymphoid population.

Histological changes were not observed in the stomach, heart, brain, and spleen of treated groups as compared to the control. The stomach and heart sections from groups CN, ANP_50_, ANP_300_, and ANP_2000_ showed the normally arranged gastric architecture and maintained polarity of myocytes arranged in muscle bundles [Figures 5A–H]. The brain from groups CN, ANP_50_, ANP_300_, and ANP_2000_ did not suffer any alteration and had normally arranged astrocytes, oligodendrocytes, axons, and interspersed capillaries in a fibrillary background [Figures 5I–L]. The spleen of groups CN, ANP_50_, ANP_300_, and ANP_2000_ showed white and red pulp with variable lymphoid population and no significant changes [Figures 5M–P].

### 3.5 Hematological and biochemical parameters

All the hematological and biochemical parameters for Group CN, ANP_50_, and ANP_300_ were under normal physiological range and did not show any significant differences compared with the control group as shown in Table 4. As all the parameters were normal, no metabolic perturbations caused by these prolonged circulating ANPs were perceived in the study. Analysis of hematological and biochemical parameters for group ANP_2000_ could not be carried out as all animals succumbed to the highest dose.

**TABLE 4:** Effect of single oral administration of different doses of *Artemisia absinthium* extract-loaded NPs on the hematological parameters, liver profile, kidney profile, and body weight of female Wistar rats after 14 days of acute oral toxicity study.

Parameters	Group CN	Group ANP_50_	Group ANP_300_
Hematological test			
Hemoglobin (Hb) (gm/dL)	14.03 ± 0.15	13.67 ± 0.25	14 ± 0.26
TLC (/cumm)	11,400 ± 818.5	13,066 ± 4,821.13	16,933.33 ± 5,839.8
Neutrophil (%)	4.17 ± 0.97	9.16 ± 2.31	7.68 ± 4.72
Lymphocyte (%)	93.23 ± 1.2	88.16 ± 3.17	88.47 ± 6.17
Eosinophil (%)	0.79 ± 0.02	1.08 ± 0.15	1.23 ± 0.55
Monocyte (%)	1.56 ± 0.21	1.86 ± 0.34	2.24 ± 1.3
Basophil (%)	0.37 ± 0.05	0.32 ± 0.19	0.39 ± 0.18
RBC (red blood cell count) (millions/cmm)	6.7 ± 0.40	6.55 ± 0.62	7.18 ± 0.70
PCV/HCT (hematocrit) (%)	47.03 ± 0.35	45.87 ± 1.2	46.3 ± 2.45
MCV (mean corpuscular volume) (fL)	68.2 ± 0.62	68.27 ± 6.67	68.57 ± 5.24
MCH (mean corpuscular Hb) (pg)	20.7 ± 0.75	20.67 ± 1.61	20.7 ± 0.79
MCHC (mean corpuscular Hb concentration) (gm/dL)	30.93 ± 0.50	30.27 ± 1.02	30.33 ± 2.1
Platelet count (lacs/cmm)	6.04 ± 0.34	8.19 ± 1.57	8.89 ± 1.29
Liver function test			
Total bilirubin (mg/dL)	0.38 ± 0.06	0.45 ± 0.20	0.45 ± 0.19
Direct bilirubin (mg/dL)	0.123 ± 0.03	0.18 ± 0.09	0.18 ± 0.1
Indirect bilirubin (mg/dL)	0.25 ± 0.04	0.26 ± 0.10	0.26 ± 0.09
SGOT (U/L)	139.37 ± 46	140.6 ± 35.69	148 ± 86.33
SGPT (U/L)	46.27 ± 9.7	45.5 ± 6.73	40.57 ± 7.81
Alkaline phosphatase (U/L)	272.17 ± 60.7	312.6 ± 125.38	194.4 ± 45.78
Total protein (gm/dL)	6.75 ± 0.10	7.09 ± 0.29	7.22 ± 0.74
Albumin (gm/dL)	3.13 ± 0.27	3.75 ± 0.29	4.03 ± 0.76
Globulin (gm/dL)	3.51 ± 0.18	3.35 ± 0.30	3.19 ± 0.51
Kidney function test			
Urea (mg/dL)	36.3 ± 6.45	33.63 ± 2.5	42.03 ± 3.02
Serum creatinine (mg/dL)	0.61 ± 0.06	0.72 ± 0.13	0.63 ± 0.07
Uric acid (mg/dL)	1.59 ± 0.31	1.98 ± 0.88	2.31 ± 1.22
Serum calcium (mg/dL)	8.64 ± 3.34	10.04 ± 2.15	9.99 ± 2.19
Sodium (mmol/L)	148.67 ± 2.30	139.33 ± 0.58	146.33 ± 9.29
Potassium (mmol/L)	4.98 ± 0.23	6.35 ± 0.30	6.47 ± 0.53
Chloride (mmol/L)	104 ± 0.58	103.67 ± 0.58	107.33 ± 2.30
Body weight (gm)			
0th day	104.33 ± 3.78	110.33 ± 1.52	126 ± 3.6
7th day	120.17 ± 3.81**	135 ± 4.58****	128.83 ± 3.25
14th day	136.17 ± 8.25**	148.33 ± 7.23*	164 ± 1.32****

Values are expressed as the mean ± SD. *p < 0.05, **p < 0.01, and ***p < 0.001 compared to the normal control group. TLC, total leucocyte count; RBC, red blood cell count; PCV/HCT, hematocrit; MCV, mean corpuscular volume; MCH, mean corpuscular hb; MCHC, mean corpuscular hb concentration; SGOT, serum glutamic-oxaloacetic transaminase; SGPT, serum glutamic pyruvic transaminase.

## 4 Discussion

The GC–MS analysis of the *A. absinthium* ethanolic extract revealed forty-five well-known terpenoids (alpha-curcumene, reynosin, phytol, isogeraniol, squalene, cycloartenol, and 24-methylenecycloartenol), phytosterols (campesterol, stigmasta-5,22-dien-3-ol, and gamma-sitosterol), flavonoids (Artemetin), and lignan (yangambin and sesartemin) that have promising antioxidant, anti-inflammatory, antimicrobial, anticancer, and antiviral properties, attributing to the medicinal potency of the plant (Gonzalez-Burgos and Gómez-Serranillos, 2012). There is no previously available information about the toxicity assessment of a single oral dose of *A. absinthium* extract-loaded NIPAAM–VP–AA polymeric NPs that can cause severe complications in rodents. In this study, we first synthesized NIPAAM–VP–AA polymeric NPs by the free-radical polymerization reaction which was further confirmed by TEM, DLS, and FTIR spectroscopy. Different concentrations of the ethanolic extract of *A. absinthium* were encapsulated into the hydrophobic core of polymeric micelles for their acute toxicological assessment as per the OECD guideline 423.

NIPAAM–VP–AA NPs loaded with a variety of therapeutic compounds (e.g., ketorolac, riluzole, and curcumene) were administered via different routes, namely, ocular, intraperitoneal, and intranasal (Gupta et al., 2000; Bisht et al., 2010; Ahmad et al., 2013; Verma et al., 2016). This is the first study, in our knowledge, that was engaged in the evaluation of the LD50 cut-off value and exposure range of these polymeric ANPs for oral administration. Different studies have reported varying LD50 values of the *A. absinthium* alcoholic extract, i.e., 2,499 mg/kg, 3,700 mg/kg, and more than 5,000 mg/kg b.wt. (Parra et al., 2001; Mahmoudi et al., 2009; Daradka et al., 2014). As per our observations and the test procedure of the OECD guideline 423, ANPs exhibit dose-dependent toxicity as all three animals died at the highest dose of 2,000 mg/kg, and their LD50 cut-off value will be 500 mg/kg body weight. According to the Globally Harmonized System, the substance having LD50 > 300–2,000 belongs to “category 4.” Thus, the evaluation of obtained data enabled us to conclude that *A. absinthium* extract-loaded NIPAAM–VP–AA polymeric NPs lie under Globally Harmonized System (GHS) “category 4,” i.e., moderately toxic. A subacute toxicity study demonstrated that orally administered poly (N-isopropyl acrylamide) copolymerized with acrylic acid (PNIPAAm-co-AAc) did not cause any toxicity in mice up to 2,000 mg/kg (Malonne et al., 2005). Therefore, we anticipated the involvement of the *A. absinthium* ethanolic extract to be responsible for the toxicity caused by ANPs at 2,000 mg/kg.

Modifications that occurred in the animal body weight during toxicity studies are reliable predictors of metabolic unfavorable effects of the test substance under investigation (da Silva Oliveira et al., 2016). There was a gradual weight gain in control as well as in animals treated at 50 mg/kg throughout the study. However, the body weight of animals treated at 300 mg/kg ANPs remained quite stagnant for up to 1 week, which significantly increased during the next week. Moreover, mild intestinal inflammation was also found in these animals, which corroborates our result, indicating that 300 mg/kg ANPs could compromise nutrient absorption and subsequent weight gain (da Silva Oliveira et al., 2018). However, this pathological and metabolic perturbation was short-lived and reversible. Similarly, behavioral symptoms of toxicity including drowsiness and loss of appetite, which were observed among these animals, also persisted for a short period. Meanwhile, there were no adverse clinical signs observed in animals treated at 50 mg/kg ANPs.

Analysis of the *in vivo* hemocompatibility of ANPs and their interaction with the cellular components of blood, namely, erythrocytes, platelets, and leukocytes, is a prerequisite to ensure its safety for effective translation to clinical implication. Assessment of hematological and biochemical parameters is crucial to monitor the toxicity profile of different chemicals which was carried out in our study to evaluate the effect of these prolonged circulating ANPs on the hematopoietic system. The hematopoietic system is extremely sensitive to toxic compounds and serves as an indicator of the physiological and pathological status of animals in toxicological studies (da Silva Oliveira et al., 2016). Once entering system circulation, polymeric micelles of amphiphilic nature with the hydrophilic outer shell (>200 nm) possess prolonged circulating time and biocompatibility by evading reticuloendothelial systems (RESs) (Liu et al., 2005; Ashraf et al., 2018). Previously, 400 nm-sized NIPAAM–VP–AA NPs showed high cytotoxicity and apoptosis in the murine macrophage cell line J774 compared to 100 nm-sized NPs, confirming macrophage activation and recruitment of the inflammatory cascade (Ashraf et al., 2018). In our study, there were no significant differences in hematological parameters among animals treated at the dose of 50 mg/kg and 300 mg/kg ANPs as compared to control after 14 days. However, there was an apparent treatment-related insignificant increase in the total leukocyte count (TLC) and platelet count in animals treated at the dose of 50 mg/kg and 300 mg/kg after 14 days of dosing. Increment in TLC indicates the activation of the immune system of animals either due to stimulated or disturbed lymphopoiesis (Çetin et al., 2010; Otuechere et al., 2014). The finding of our study with minor increment in TLC and platelet count suggests immunomodulatory and wound healing properties of *A. absinthium* extract-loaded NPs at lower doses (Yamthe et al., 2012), which is in agreement with the previous study of Amat et al. that demonstrated immunomodulatory effects of *A. absinthium* extracts in mice indicating toward its antiradical and antioxidant activity (Amat et al., 2010). Therefore, we suggest that ANPs with 100–200 nm hydrodynamic size are hemocompatible and do not cause lethal alterations in hematological parameters at a single low dose. Liver- and kidney-related biochemical enzymes are reliable indicators of possible toxicity caused by exposure to different drugs (Farag et al., 2006). All the liver- and kidney-related enzymes, as well as electrolyte levels, were normal in animals treated at the dose of 50 mg/kg and 300 mg/kg ANPs. Therefore, we suggest that neither metabolic disturbance nor renal dysfunction was caused by ANPs at low doses in the rats. However, the impact of 2,000 mg/kg ANPs on hematological and biochemical parameters could not be inferred as all the animals died during the experiment.

In our study, histological evidence reveals the toxic potential of ANPs mainly in the intestine and kidney. Orally administered small NPs (50–100 nm) can move through the intestinal epithelium to lamina propria of the GI tract via a transcellular pathway comprising enterocytes to finally enter the systemic circulation, while large NPs (>100 nm) are taken up by M cells (Pridgen et al., 2015; Reinholz et al., 2018). It is also reported that NPs may experience direct and indirect movement in systemic circulation through the intestinal lymphatic system and hepatic portal system, respectively (Hua, 2020). We observed that a single oral dose of ANPs at 300 mg/kg caused mild intestinal inflammation, while 2,000 mg/kg caused ulceration of intestinal mucosa and inflammatory response in the lamina propria which, in turn, indicates that ANPs increased gut permeability in rats. Additionally, adverse clinical signs, including diarrhea and loss of appetite, were seen in group ANP _2000_, which was consistent with our finding of intestinal ulceration and inflammation, indicating disturbed intestinal function accompanied by diarrhea at a high dose of ANPs (Wang et al., 2019). Necropsy analysis of animals treated at 2,000 mg/kg also revealed the deposition of a small amount of ANPs in the lumen of the intestine. These findings can be correlated with an *in vitro* release study that reported a remarkable increment in LCST of the pH- and temperature-responsive, insulin-loaded NIPAAM and AA derivative-based hydrogels under the condition of neutral artificial intestinal fluid (pH 6.8), resulting in hydrogel swelling and fast release of insulin (Gao et al., 2013). Thus, we can anticipate that ANPs would also have experienced an increase in LCST (>37°C) at intestinal pH, eventually collapsing and releasing its content. However, our study cannot infer the amount of ANPs collapsing at intestinal pH and the amount of ANPs entering the systemic circulation and eventually getting eliminated from the body.

Several studies suggested that NPs entering systemic circulation are further distributed to different organs triggering further pathological changes (Chen et al., 2006). In this study, the liver of rats from all the groups showed dilatation of sinusoids and few central veins with interspersed congested blood vessels as a functional consequence of impaired blood flow. The kidney is the blood-filtering organ that receives a major portion of the systemic circulation; thus, direct exposure and accumulation of NPs can be responsible for renal tissue rupture or hemorrhage. In this study, the kidneys of animals treated at 300 mg/kg and 2,000 mg/kg showed focal areas of hemorrhage in the renal cortex without traces of ischemia, necrosis, or any other structural disintegration. The kidneys of animals treated at 50 mg/kg were also histopathologically normal with focal areas of inflammatory infiltrates in the cortex. Thus, we can postulate that these morphological and structural changes were associated with the fact that ANPs were able to pass through the intestinal epithelium and reached the lamina propria through which they are phagocytosed and transported to the kidney causing acute renal injury. These pathological changes due to a single oral dose persisted among animals treated at 50 mg/kg and 300 mg/kg ANPs even after 14 days of acute exposure advocate toward their capacity to cause moderate-to-mild renal toxicity. However, no pathological damage or toxicity of these ANPs was found in the stomach, heart, brain, or spleen. Therefore, the *in vivo* behavior, role of hydrodynamic size, nature of interaction with the intestinal tract, and fate in the GI tract require further investigation.

## 5 Conclusion

The findings of our study demonstrate that *A. absinthium* extract-loaded NIPAAM–VP–AA polymeric NPs (ANPs) have LD50 cut-off value equal to 500 mg/kg body weight and belong to ‘category 4’ of the GHS, with moderately toxic nature as high dose caused intestinal and renal injury in rats. Our preliminary study exhibits limitations being only concerned with the assessment of the LD50 cut-off value of ANPs, although providing a new insight into the understanding of immunostimulation by ANPs and emphasizing the need of investigating the hemocompatibility and chronic toxicity of ANPs for clinical application. However, an appropriate dose of these NPs presents a promising site-specific drug delivery system that could be employed against different medical disorders.

## Data Availability

The original contributions presented in the study are included in the article/Supplementary Material. Further inquiries can be directed to the corresponding author.

## Abbreviations

GC–MS, gas chromatography–mass spectrometry; NIPAAM, N-isopropyl acrylamide; LCST, lower critical solution temperature; VP, N-vinyl pyrrolidone; AA, acrylic acid; ANPs, *Artemisia absinthium* whole extract-loaded (NIPAAM–VP–AA) polymeric nanoparticles; TEM, transmission electron microscopy; DLS, dynamic light scattering; FT-IR, Fourier-transform infrared spectrophotometry; PDI, polydispersity index; OECD, Organization for Economic Co-operation and Development; and GHS, Globally Harmonized System.
